# Case report: Unveiling the silent threat in the ICU – a case of disseminated invasive aspergillosis in a patient with fulminant myocarditis

**DOI:** 10.3389/fimmu.2024.1481335

**Published:** 2024-11-07

**Authors:** Yimin Xue, Jibin Mo, Kun Cheng, Ying Xue, Dongjie Chen, Fenghui Lin, Han Chen

**Affiliations:** ^1^ Fourth Department of Critical Care Medicine, Shengli Clinical Medical College of Fujian Medical University, Fujian Provincial Hospital, Fuzhou University Affiliated Provincial Hospital, Fuzhou, Fujian, China; ^2^ Department of Ophthalmology, Shengli Clinical Medical College of Fujian Medical University, Fujian Provincial Hospital, Fuzhou University Affiliated Provincial Hospital, Fuzhou, Fujian, China; ^3^ Clinical Microbiology Laboratory, Shengli Clinical Medical College of Fujian Medical University, Fujian Provincial Hospital, Fuzhou University Affiliated Provincial Hospital, Fuzhou, Fujian, China

**Keywords:** invasive aspergillosis, disseminated infection, immunocompromise, PET/CT, mNGS

## Abstract

Invasive aspergillosis (IA) significantly increases mortality in critically ill patients in the ICU and its occurrence is closely related to immunocompromise. Dissemination of IA is easily misdiagnosed and mistreated due to its ability to invade multiple systems throughout the body and lack of typical clinical manifestations. In this case, a 25-year-old previously healthy woman was hospitalized with fulminant myocarditis and treated with veno-arterial extracorporeal membrane pulmonary oxygenation (VA-ECMO) support and intravenous acyclovir, high-dose methylprednisolone, and immunoglobulin. 6 days later, she was successfully weaned from VA-ECMO and underwent cardiac rehabilitation. On day 10, she developed a fever (Tmax 38.3°C) and an irritating cough and began to experience reduced vision over the right eye with eye pain, redness, photophobia, and tearing 2 days later. Administration of levofloxacin eye drops and tobramycin/dexamethasone eye ointment was ineffective. The patient was positive for serum *Aspergillus* galactomannan antigen. Positron emission tomography/computed tomography (PET/CT) scan showed multiple hypermetabolic cavitary nodules in both lungs (SUVmax3.6) and thickening of the ocular ring wall with hypermetabolism in the right eye (SUVmax3.2). Ophthalmologic examination revealed that her best-corrected visual acuity in the right eye was reduced to light perception with an intraocular pressure of 21 mmHg, and B-scan ultrasonography showed vitreous opacity and retinal edema with mild detachment in the right eye. Metagenomic next-generation sequencing (mNGS) identified a large number of *Aspergillus fumigatus* sequences in bronchoalveolar lavage fluid, blood, and aqueous humor from the right eye, supporting the diagnosis of pulmonary and ocular involvement due to disseminated IA. Vitrectomy, anterior chamber irrigation, combined with intravenous and intravitreal injections of voriconazole and liposomal amphotericin B eventually cured the patient. This case highlights the importance of early identification and intervention regarding disseminated IA in immunocompromised critically ill patients, especially in the presence of multiple organ involvement.

## Introduction

*Aspergillus* species are a large group of common saprophytic fungi that occur widely in the natural environment. The lungs are the target organ most frequently infected by *Aspergillus*. Inhalation of *Aspergillus* spores or conidia may lead to a variety of lung diseases, including invasive pulmonary aspergillosis (IPA), allergic bronchopulmonary aspergillosis (ABPA), pulmonary aspergilloma (PA), and chronic necrotizing pulmonary aspergillosis (CNPA), depending essentially on the host’s immunological status (1). In immunocompromised patients, aspergillosis has a highly aggressive and invasive clinical feature, with the potential to cause severe disseminated infections in multiple organs throughout the body (2). The frequency of invasive aspergillosis (IA) in critically ill patients is increasing even in the absence of an apparent susceptible immunodeficiency, which is concerning given its association with high mortality (3, 4). Currently, the diagnostic standard for IA is based on histological documentation of typical hyphae and a positive *Aspergillus* culture (5). However, the definitive diagnosis is often delayed due to the diversity and non-specificity of the clinical manifestations of IA, especially in the presence of multiple organ involvement. Herein, we report a rare case of disseminated IA in a patient with fulminant myocarditis (FM) to improve clinicians’ knowledge regarding this disease.

## Case presentation

A 25-year-old previously healthy woman was admitted to Fujian Provincial Hospital (Fujian, China) with fever (Tmax 39.2°C) and chest pain for 3 days. She had a cold accompanied by fatigue, headache, and loss of appetite 5 days earlier, which was slightly relieved by self-administration of acetaminophen (500-1000 mg/day). She denied any history of previous heart disease, hypertension, diabetes, or hyperlipidemia. Chest pain worsened 6 hours before admission with malaise, nausea, vomiting, and progressive shortness of breath. Shortly, she developed respiratory failure and hemodynamic instability and was transferred to the ICU for mechanical ventilation and aggressive supportive care. Further workup revealed markedly elevated cardiac troponin I (cTnI, 101.30 ng/mL) and N-terminal pro-brain-type natriuretic peptide (NT-proBNP, 28283.00 pg/mL) levels, diffusely decreased left and right ventricular systolic function (LVEF and RVEF of 10% and 7%, respectively), and persistent ventricular tachycardia, consistent with FM. Due to severe cardiogenic shock, the patient was placed on veno-arterial extracorporeal membrane oxygenation (VA-ECMO) support and treated empirically with intravenous acyclovir (500 mg/day for 5 consecutive days), high-dose methylprednisolone (200 mg/day for 5 consecutive days), and immunoglobulin (20 g/day for 5 consecutive days). Additionally, serum inflammatory markers, including white blood cell (WBC) count (16.90 × 10^9^/L), C-reactive protein (CRP, 80.21 mg/L), and procalcitonin (PCT, 11.42 ng/mL), were found to be significantly elevated. Other bloodwork revealed total bilirubin (TBil) of 14.12 μmol/L, alanine aminotransferase (ALT) of 1833 U/L, aspartate aminotransferase (AST) of 2824 U/L, γ-glutamyl transferase (GGT) of 184 U/L, blood urea nitrogen (BUN) of 26.8 mmol/L, creatinine (Cr) of 235 μmol/L, prothrombin time (PT) of 33.1 s, international normalized ratio (INR) of 3.03, activated partial thromboplastin time (APTT) of 56.4 s, fibrinogen (Fib) of 0.63 g/L, and platelet (PLT) count 29 × 10^9^/L, indicating multiple organ dysfunction. Given the high risk of secondary bacterial infections due to the patient’s critical condition and the presence of multiple invasive devices, she received broad-spectrum intravenous antibiotic therapy with 1.0 g meropenem q8h and 1.0 g vancomycin q12h. Thereafter, the patient’s cardiac function gradually recovered (cTnI decreased to 7.41 ng/mL, NT-proBNP decreased to 7282 pg/mL, and LVEF and RVEF increased to 40% and 20%, respectively, on day 6), and other organ functions gradually improved (ALT decreased to 531 U/L, AST decreased to 163 U/L, GGT decreased to 81 U/L, BUN decreased to 13.1 mmol/L, Cr decreased to 138 μmol/L, and PLT count increased to 73 × 10^9^/L, and other coagulation parameters showed no significant abnormalities on day 6). Therefore, VA-ECMO support was stopped on day 6, tracheal intubation was removed after 2 days, and cardiac rehabilitation was applied. During the above treatment period, the patient had no fever, and her inflammatory markers gradually normalized (WBC count decreased to 9.1 × 10^9^/L, CRP decreased to 6.45 mg/L, and PCT decreased to 0.56 ng/mL on day 7). Meropenem and vancomycin were discontinued on day 8 since no positive bacteria were found in repeated blood, sputum, and urine cultures. The antibiotic regimen was finally changed to intravenous ceftazidime (2.0 g q12h). Methylprednisolone was reduced to 100 mg/day on day 6, halved every 3 days until reduced to 12.5mg/day, and then discontinued.

**Figure 1:**
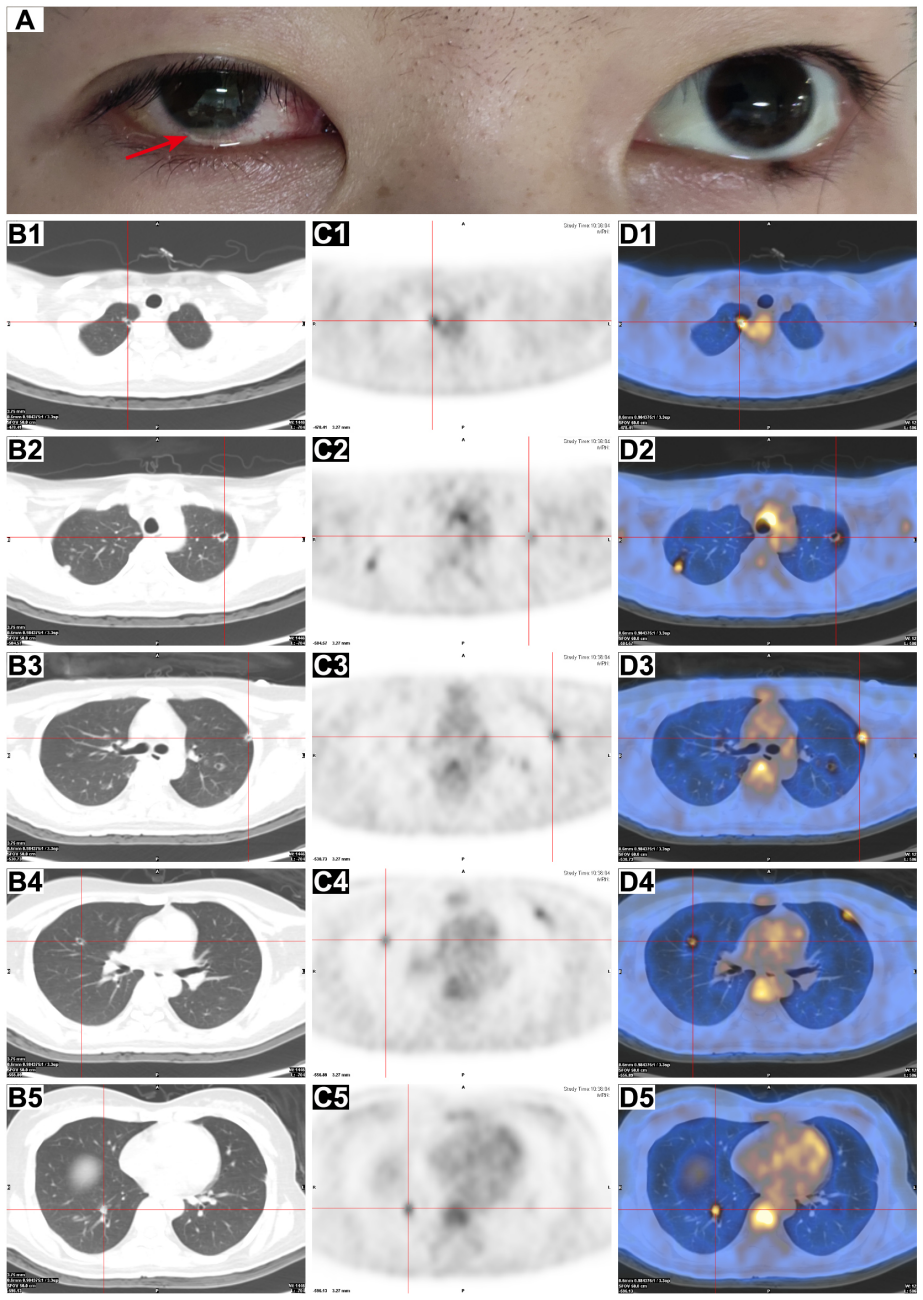
Ocular appearance and PET/CT scan of the chest. **(A)** Clinical photograph of the patient on day 13 after admission, showing conjunctival congestion in the right eye with pus in the anterior chamber (red arrow). **(B–D)** Axial chest CT **(B1–B5)**, PET **(C1–C5)**, and fusion PET/CT **(D1–D5)** images showed the presence of multiple cavitary nodules in both lungs with increased FDG uptake (red crosses).

**Figure 2:**
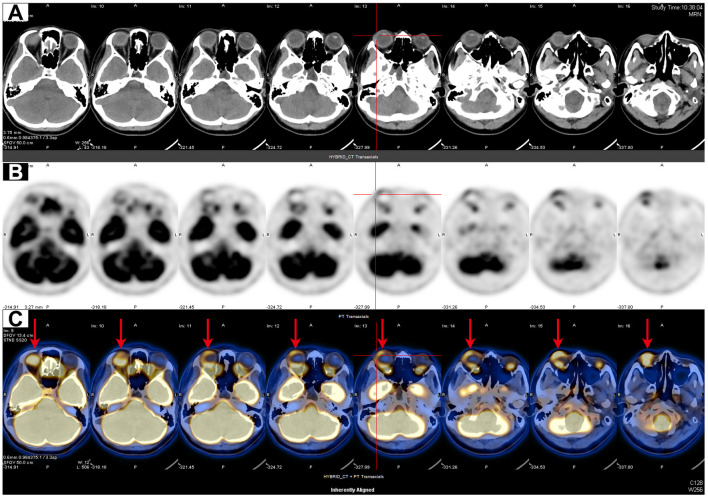
PET/CT scan of the orbit. **(A–C)** Axial orbital CT **(A)**, PET **(B)**, and fusion PET/CT **(C)** images showed thickening of the ocular ring wall with increased FDG uptake in the right eye (red arrows).

**Figure 3:**
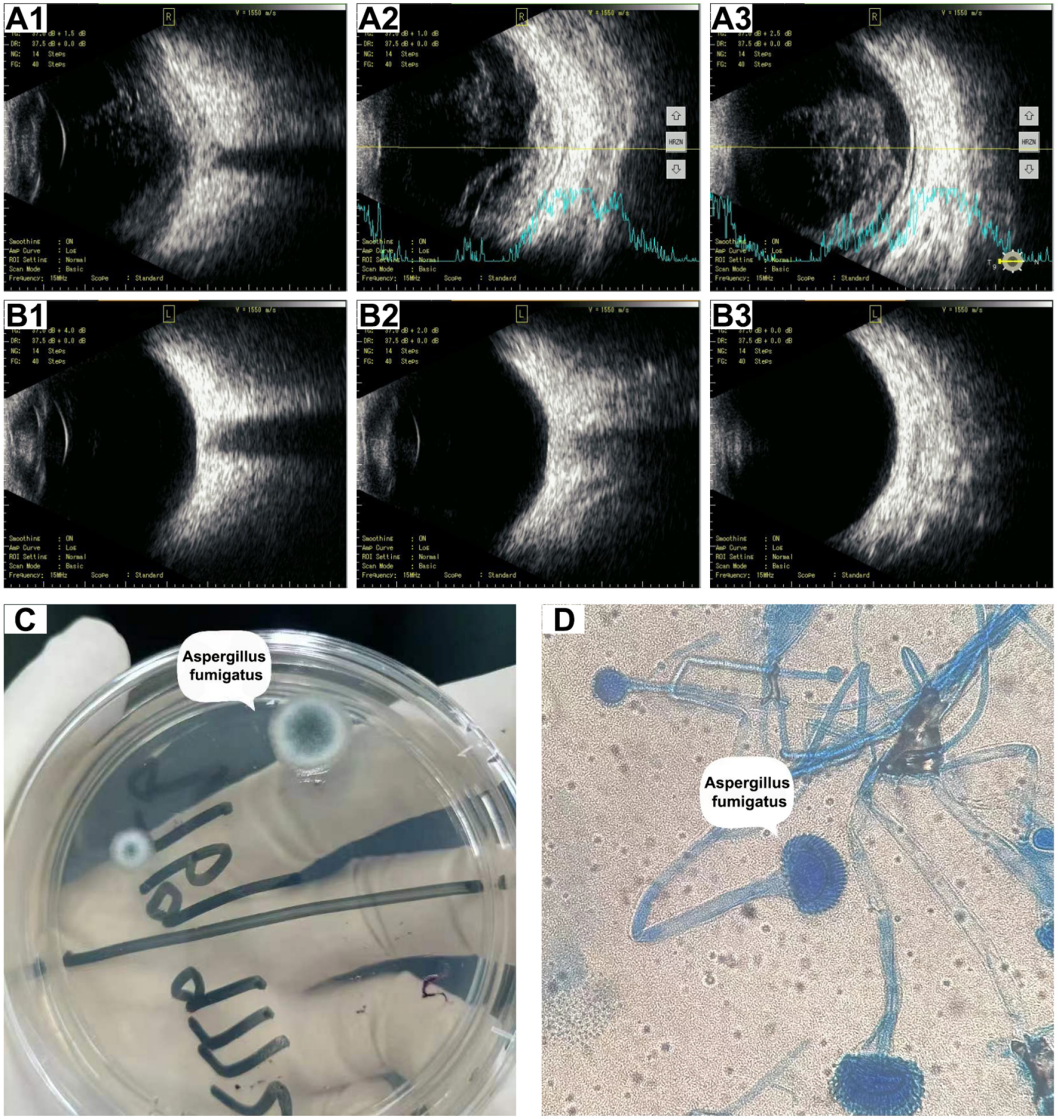
Ocular B-scan ultrasonography and culture of right aqueous humor before treatment. **(A)** B-scan ultrasonography images **(A1–A3)** of the right eye showed vitreous opacity and retinal edema with mild detachment. **(B)** B-scan ultrasonography **(B1–B3)** of the left eye revealed no abnormalities. **(C)** Potato dextrose agar medium showing the colonies of *A*. *fumigatus*. **(D)** Microscopic examination showing typical *A*. *fumigatus* morphology including branching hyphae and granular spores (Lactophenol cotton blue staining, 1000×).

**Figure 4:**
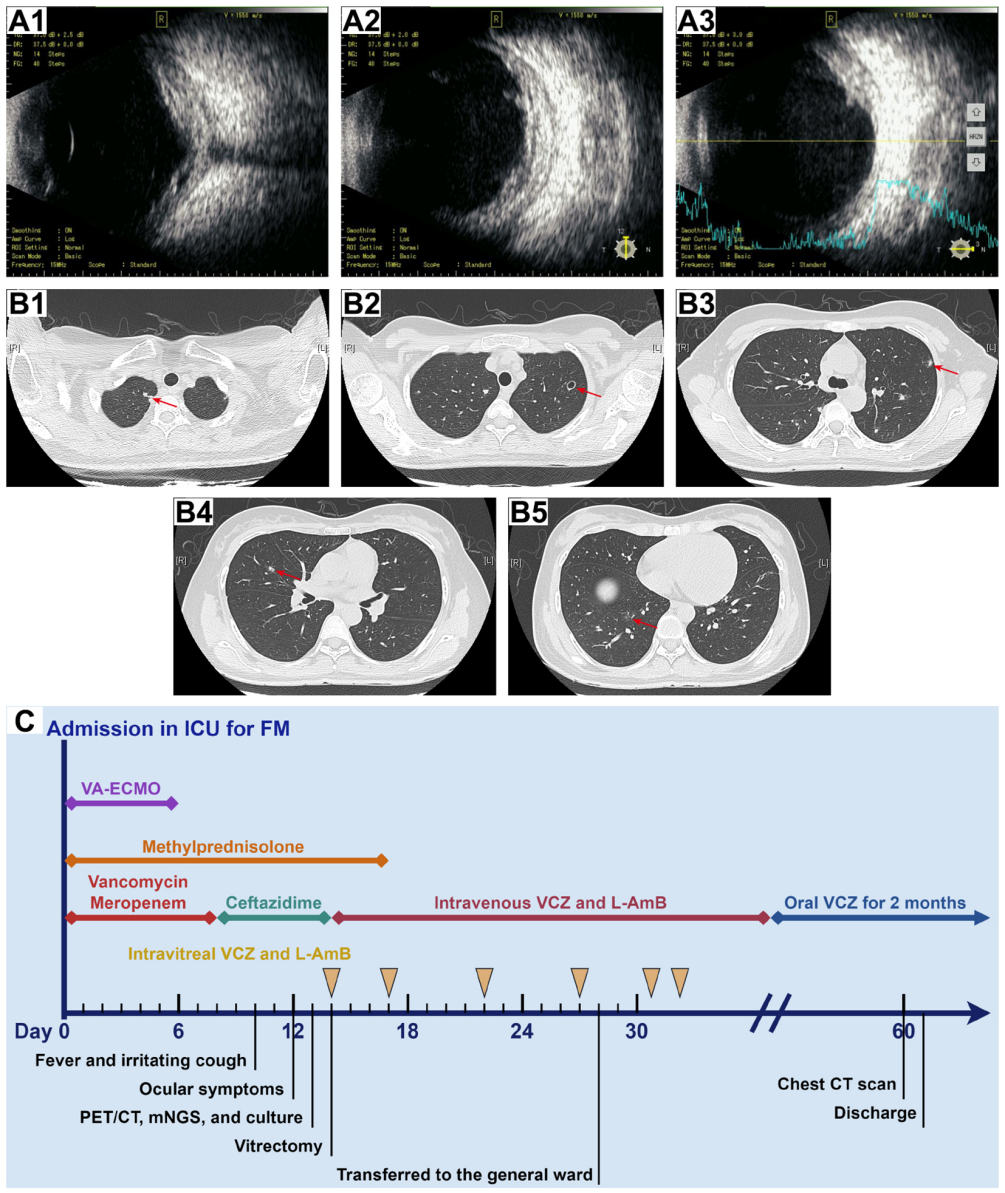
B-scan ultrasonography of the right eye and chest CT scan after treatment and timeline of the hospitalization process. **(A)** B-scan ultrasonography of the right eye on day 28 **(A1–A3)** revealed a significant reduction in vitreous opacity, while the retina remained edematous with mild detachment. **(B)** Chest CT scan on day 60 **(B1–B5)** showed significant absorption of *Aspergillus* lesions in both lungs (red arrows). **(C)** The timeline and treatment course for the patient.

On day 10, she developed a fever (Tmax 38.3°C) and an irritating cough and began to experience reduced vision over the right eye with eye pain, redness, photophobia, and tearing 2 days later. The patient continued her original intravenous antibiotic regimen with the addition of levofloxacin eye drops (one drop six times daily to the right eye) and tobramycin/dexamethasone eye ointment (once a night before bed) for ocular inflammation. The patient’s eye pain gradually worsened and she presented with blurred vision in her right eye (**Figure 1A**). Laboratory tests revealed re-elevated serum inflammatory markers (WBC count of 15.70 × 10^9^/L, CRP of 17.10 mg/L, and PCT of 1.13 ng/mL), positive serum galactomannan *Aspergillus* antigen (2.63, cut-off value of 0.50), and decreased levels of CD3 cells (422/μL), CD4 cells (221/μL), CD8 cells (188/μL), NK cells (20/μL), CD19 cells (49/μL), CD45 cells (505/μL), complement C3 (0.718 g/L), and complement C4 (0.069 g/L), suggesting that the patient was immunocompromised and might have IA. Subsequently, the patient underwent a whole-body positron emission tomography/computed tomography (PET/CT) for *Aspergillus* dissemination evaluation. The results showed multiple hypermetabolic cavitary nodules in both lungs (SUVmax3.6, **Figures 1B–D**) and thickening of the ocular ring wall with hypermetabolism in the right eye (SUVmax3.2, **Figures 2A–C**). No abnormal accumulation was found in other organs. Upon ophthalmologic examination, her best-corrected visual acuity (BCVA) in the right eye was reduced to light perception with an intraocular pressure (IOP) of 21 mmHg, while her BCVA in the left eye was 1.0 with an IOP of 14 mmHg. The ocular B-scan ultrasonography showed vitreous opacity and retinal edema with mild detachment in the right eye (**Figure 3A**). Examination of the left eye did not reveal any abnormalities (**Figure 3B**). To further confirm the diagnosis of IA, bronchoalveolar lavage fluid, blood, and aqueous humor from the right eye were also retained for metagenomic next-generation sequencing (mNGS) and culture. mNGS identified a large number of *Aspergillus fumigatus* (*A. fumigatus*) sequences in the three samples described above, supporting the diagnosis of pulmonary and ocular involvement due to disseminated IA. Cultures of bronchoalveolar lavage fluid and aqueous humor from the right eye were consistent with mNGS (**Figures 3C, D**), but blood culture was negative. Ceftazidime was then discontinued and intravenous voriconazole (VCZ) 4.0 mg/kg q12h after a 6.0 mg/kg q12h loading dose and liposomal amphotericin B (L-AmB) 1.0 mg/kg daily were initiated on day 14. The patient also underwent vitrectomy, anterior chamber irrigation, and intravitreal injections of VCZ (100 μg/0.1 mL) and L-AmB (5 μg/0.1 mL) at the same time. A total of six intravitreal injections were performed (each 3-5 days apart), and the patient’s ocular pain was significantly relieved. Sequential therapy with oral VCZ (200 mg bid) was applied for two months following a one-month course of intravenous antifungal therapy. On day 28, B-scan ultrasonography of the right eye showed that the vitreous opacity was significantly relieved, but the retinal edema with mild detachment still existed (**Figure 4A**). She was then transferred to the general ward for rehabilitation and continued to receive regular follow-up in the ophthalmology outpatient clinic. On day 45, after one month of antifungal treatment, the patient experienced a slight recovery of vision, with the right eye BCVA improving to counting fingers at 30 cm and IOP decreasing to 15 mmHg. On day 60, a chest CT examination showed significant absorption of the pulmonary aspergillosis lesions (**Figure 4B**), and the patient was discharged on day 62 (**Figure 4C**). At the one-month follow-up after discharge, the patient exhibited no significant reduction in visual acuity, with a BCVA of counting fingers at 50 cm and a stable IOP of 13 mmHg in the right eye. Additionally, inflammation was well-controlled, and there was no evidence of systemic fungal infection.

## Discussion

Fungal infections, particularly those caused by *Candida* and *Aspergillus* species, can be life-threatening in critically ill patients with heart disease. These infections are more common in immunocompromised individuals, including those who have received heart transplants, those with immune-mediated myocarditis, and those who have undergone cardiac surgery (6–8). Effective management requires a multidisciplinary approach, involving prevention strategies, early diagnosis, and appropriate antifungal therapy (9). FM usually results from a cardiotropic viral infection, followed by severe immune-mediated inflammatory destruction of the myocardium (10). A life support-based comprehensive treatment regimen, including antiviral agents, glucocorticoids, immunoglobulins, and mechanical circulatory support systems, can reduce massive cardiomyocyte death, thereby improving the survival of FM patients (11). In this case, we successfully helped the patient through the cardiogenic shock phase. However, severe viral infections, high doses of corticosteroids, immunocompromise, and extensive invasive procedures and devices increased this patient’s susceptibility to disseminated IA (12, 13). The portal of entry for *Aspergillus* invasion was presumably the central venous catheter, which compromised the integrity of the cutaneous barrier and allowed the *Aspergillus* to invade systemic circulation, leading to disseminated infection. Therefore, aseptic techniques during procedures, along with vigilant monitoring, as well as the timely removal of unnecessary devices, are crucial for the prevention of IA in the ICU.

It is well known that earlier treatment is associated with better survival, but timely and accurate diagnosis of IA is difficult. Galactomannan is a major component of the *Aspergillus* cell wall, and detection of galactomannan antigen release may be useful for screening high-risk patients and evaluating therapy effectiveness (14). The sensitivity of this method is limited, ranging from 30-46%, and it is prone to false-positive results due to cross-reactivity with other bacterial components or semi-synthetic penicillin (15, 16). Therefore, further imaging evaluation before etiological results is essential when IA is suspected. Conventional CT imaging is typically beneficial for diagnosis, but radiological features of aspergillosis are nonspecific, and the CT finding known as the “halo-signal” is not always clearly detectable (17). PET/CT is a functional imaging modality that depicts the intensity of glucose metabolism across different body regions using radioisotope 18F labeled fluorodeoxyglucose (FDG). When activated, inflammatory cells release large amounts of pro-inflammatory cytokines, and the expression of glucose transporters is upregulated, promoting the uptake of 18F-FDG by these inflammatory cells (18). Studies utilizing 18F-FDG uptake in inflammatory settings have become a field of increasing interest in the last few years (19). Compared to alternative nuclear medicine imaging techniques, PET/CT allows precise spatial localization of 18F-FDG distribution based on anatomical structures, thus providing a consistent and reliable method for early diagnosis of IA and detection of extrapulmonary involvement (20). Reports in the literature indicate that *Aspergillus* nodules have variable metabolic activities (low to high 18F-FDG uptake) depending on their aggressiveness and morphology (20, 21). Different metabolic imaging manifestations of *Aspergillus* infection have been categorized: cold nodules, with discrete peripheral metabolism; iso-metabolic nodules, with 18F-FDG uptake similar to or lower than the mediastinal blood pool; and hypermetabolic nodules, with 18F-FDG uptake higher than the mediastinal blood pool, as in our patient (22). Clinicians should incorporate PET/CT into the diagnostic and therapeutic management of patients with suspected IA to provide a thorough evaluation and guide the choice of the most appropriate therapy. By PET/CT scan, we determined that the organs affected by *Aspergillus* invasion were limited to the lungs and the right eye.

*Aspergillus* disseminates hematogenously, potentially forming small emboli that can occlude pulmonary microvasculature, thereby inducing ischemia and necrosis in the affected tissues. This pathological process facilitates fungal proliferation and culminates in the development of multiple randomly distributed nodular and cavitary lesions within the pulmonary parenchyma. When *Aspergillus* reaches the eyes, it may cross the blood-ocular barrier to enter the internal ocular spaces, leading to endogenous Aspergillus endophthalmitis (EAE), a rare vision-threatening disease with an appreciable mortality rate (23). In our case, the patient had no history of ocular trauma or surgery, so we speculated that it was likely that immunosuppression during FM therapy significantly disrupted the patient’s immune homeostasis and increased blood-ocular barrier permeability, allowing *Aspergillus* to spread via the bloodstream to her right eye. The concordance of mNGS results of bronchoalveolar lavage fluid, blood, and right aqueous humor supported this diagnosis, although blood cultures were negative. It is important to note that *A. fumigatus* has a greater propensity to invade the eyes than other *Aspergillus* species and may cause severe damage to ocular tissues (24). Moreover, some cases of EAE have also been reported in immunocompetent patients (25). Sastry et al. (26) reported a case of EAE in an immunocompetent patient secondary to pulmonary changes that occurred from previously treated tuberculosis. Therefore, the possibility of EAE should be considered in patients with sudden onset of eye pain, decreased vision, and no history of ocular injury, and a thorough systemic examination is necessary to clarify the extent of infection dissemination and identify the pathogen.

The application of mNGS played a decisive role in the rapid diagnosis of this patient. Compared to culture-based methods, mNGS technology shows significant advantages in terms of detection efficiency and speed (27). Diagnostic efficiency analysis revealed that mNGS had 88% sensitivity and 100% specificity for endophthalmitis diagnosis (28). In addition, mNGS is less affected by antibiotics and can even detect fragments of killed microorganisms, providing a basis for clinical antibiotic selection, which reflects its characteristics of high-throughput and unbiased detection (29). Zhu and colleagues reported the positive rates of mNGS and culture for detecting endophthalmitis pathogens were 88.89% and 27.78%, respectively (30). However, mNGS does not provide information on drug susceptibility. Combining mNGS with culture should be preferred to help accelerate clinical decision-making and establish pathogen-oriented therapeutic approaches.

The main treatment options for this patient included systemic and intravitreal antifungal medications combined with vitrectomy. The broad-spectrum antifungal triazole VCZ has been approved as a first-line agent for the initial treatment of IA, with L-AmB as an alternative. Combination therapy may be considered to improve outcomes and survival in immunocompromised, critically ill IA patients with multiple sites of involvement, as in our patient (31). Several studies have confirmed the synergistic effect of azoles with L-AmB, the mechanism involved may be the increased permeability to azoles and the enhanced inhibition of fungal ergosterol synthesis when exposed to both antifungal drugs simultaneously, resulting in greater antifungal activity (32). Aoki et al. (33) found that the combination of VCZ and L-AmB may be beneficial in stabilizing IA in patients undergoing hematopoietic stem cell transplantation. In a mouse model of experimental central nervous system aspergillosis, a low dose (1 mg/kg) of L-AmB in combination with VCZ significantly reduced intracranial and renal *Aspergillus* loads and improved mouse survival compared to the monotherapy group (34). High-dose L-AmB combination therapy may increase toxicity to the organism, which is a risk factor to be alerted to in our patient as her organ function had just recovered. Intravitreal injection of antifungal agents, including VCZ and L-AmB, is a key method to achieve high intraocular drug concentrations while reducing drug-related systemic toxicity. Intravitreal injection of VCZ is effective in the treatment of EAE, but it has a significantly shorter half-life in vitrectomized eyes (approximately 2.5 hours), and in combination with intravitreal L-AmB (half-life of approximately 1.8 days) can help maintain therapeutic intraocular concentrations based on systemic administration and reduce the side effects associated with repeated intravitreal injections (35, 36). No clear consensus exists on the indication and timing for vitrectomy in the management of EAE. Prompt vitrectomy is recommended in severe cases complicated by marked vitreous opacity, retinal detachment, poor initial visual acuity, and more virulent pathogens (37). Early vitrectomy with improved visual outcomes has been noted in recent studies (38, 39). Vitrectomy usually serves both therapeutic and diagnostic functions. Vitreous removal reduces the fungal load and accumulation of inflammatory factors, enhances antifungal efficiency by removing the vitreous cavity segregation, and allows access to specimens for culture and drug sensitivity testing. In our case, vitrectomy accelerated the clearance of *A. fumigatus* and limited retinal injury caused by EAE, thereby avoiding complete retinal detachment.

## Conclusion

We present a rare case of disseminated IA in a patient with FM. Currently, the patient is recovering well, with no signs of recurrence of the Aspergillus infection. Her visual acuity is stabilized at the level of counting fingers and continues to be followed up regularly in the ophthalmology clinic. The clinical manifestations of disseminated IA can be variable, and therefore constant vigilance is necessary, especially in managing immunocompromised critically ill patients. Effective preventive measures, timely and accurate diagnosis, and prompt treatment are crucial for the prognosis of the disease.

## Data Availability

The original contributions presented in the study are included in the article/supplementary material. Further inquiries can be directed to the corresponding author.
